# Displacement Mapping as a Highly Flexible Surface Texturing Tool for Additively Photopolymerized Components

**DOI:** 10.3390/mi15050575

**Published:** 2024-04-26

**Authors:** Robert Bail, Dong Hyun Lee

**Affiliations:** 1 Graduate School of Convergent Systems Engineering, Dankook University, Yongin 16890, Republic of Korea; 2 Department of Polymer Science and Engineering, Dankook University, Yongin 16890, Republic of Korea

**Keywords:** mesh modeling, resin printing, surface finish, random texture, nature-inspired surfaces

## Abstract

Displacement mapping is a computer graphics technique that enables the design of components with regularly or randomly textured surfaces that can be quickly materialized on a three-dimensional (3D) printer when needed. This approach is, in principle, more flexible, faster, and more economical compared to conventional texturing methods, but the accuracy of the texture depends heavily on the parameters used. The purpose of this study is to demonstrate how to produce a surface-textured part using polygonal (mesh) modeling software and a photopolymerizable resin and to develop a universal methodology to predict the dimensional accuracy of the model file log combined with a resin 3D printer. The printed components were characterized on a scanning confocal microscope. In the setup used in this study, the mesh size had to be reduced to 10% of the smallest feature size, and the textured layer had to be heavily (×4.5) overexposed to achieve the desired accuracy. As a practical application, two functional stamps with a regular (honeycomb) and a random texture, respectively, were successfully manufactured. The insights gained will be of great benefit for quickly and cost-effectively producing components with innovative patterns and textures for a variety of hobby, industrial, and biomedical applications.

## 1. Introduction

In science and engineering, a texture describes the topography of a part surface in terms of roughness, waviness, and lay [1]. Textured surfaces can help improve the functionality of a part and thereby create added value, for example, by improving its aesthetic, mechanical, tribological, thermodynamic, optical, acoustic, wetting, or bioactive properties [2]. With the advent of three-dimensional (3D) printing and associated 3D modeling software, surface texturing can be approached as an integral part of the design and manufacturing process, i.e., it is possible to create literally any surface texture at the same time as the component is made [3]. This approach is more flexible, faster, and more cost-effective compared to traditional texturing methods like hot embossing [4], micromilling [5], laser surface texturing [6,7], or soft lithography [8]. Moreover, it allows textures to be fabricated with perfect uniformity and on curved surfaces, and even textures with overhangs or undercuts can be created with relative ease [9].

Three-dimensional modeling can be divided into parametric and polygonal techniques. Parametric 3D modeling is very precise and ideal for most engineering and architectural applications because the modeled objects are described using input parameters that are fed into mathematical algorithms [10]. However, this approach reaches its limits with organic shapes or complex textures that are difficult to describe using parameters and algorithms. In polygonal 3D modeling, in contrast, an object is constructed from polygons, i.e., flat faces with at least three corners, which is very versatile and therefore more suitable for organic models, photorealistic scenes, and also textures [11]. However, the objects created in this way can be less accurate due to the lack of precise parameters, and high mesh resolution can place high demands on computer hardware [12]. Among the various 3D printing techniques, vat photopolymerization processes (3D resin printing) like stereolithography (SLA), digital light processing (DLP), or liquid crystal display (LCD) mask printing are rather suitable for incorporating surface texture since their resolution is high enough to accurately reproduce features of well under 100 μm at a reasonable cost [13].

Given the great importance that decorating virtual objects has in the animation industry, polygonal modeling programs are usually equipped with a range of different 2D texturing tools [14]. Unlike many other techniques, such as bump mapping, which merely creates the illusion of a texture by changing pixel shades, with displacement mapping, the geometric position of individual vertices of the respective parts of a mesh is actually displaced, usually in the direction of the local surface normal [15], resulting in a truly three-dimensional surface topology that actually occurs on a 3D printed part. The difference between a textured and a non-textured surface in the normal direction can be described by the equation

(1) $$\vec{r}\left(u,\;v\right)=\vec{p}\left(u,\;v\right)+\vec{N}(u,\;v)\vec{h}(u,\;v),$$

where the textured surface (mesostructure) is denoted by $\vec{r}\left(u,\;v\right)$, the non-textured mesh surface (macrostructure) is represented by $\vec{p}\left(u,\;v\right)$, $\vec{N}(u,\;v)$ is the unit normal of the macrostructure surface, $\vec{h}(u,\;v)$ is the displacement described by a gray scale height map, and the parameters u and v are the texture coordinates [16]. Optionally, the height map can be superimposed with a color map to obtain a highly photorealistic look on a display or monitor [17].

The combination of polygonal 3D modeling, displacement mapping, and 3D resin printing represents a rapid and highly flexible texturing tool. However, it is not routinely used in science and engineering, partly due to its origins in computer graphics, but also due to concerns about the accuracy of the generated parts and surfaces. This study presents an innovative method to characterize the dimensional capability of the model file protocol and subsequent 3D printing. Applying this method, we demonstrated that a protruding texture was reproduced with high accuracy when the resolution of the mesh model was at least ten times higher than the desired feature size and the textured surface layers were significantly overexposed. We hope that in the future, displacement mapping will be used more extensively in the design and small-scale production of micro-textured components for a wide range of hobbyist, industrial, and biomedical applications.

## 2. Materials and Methods

**Figure 1:**
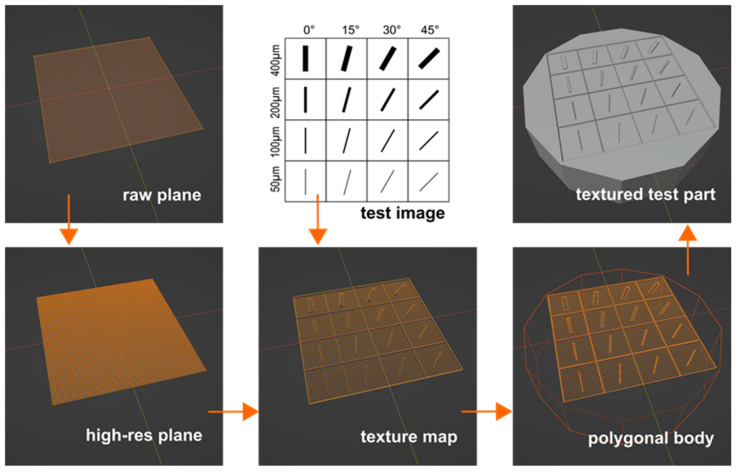
Process of digitally generating a textured surface using mesh modeling software.

A test image was created in an open-source vector graphics editor (Inkscape, ver. 1.2.2). The image showed a 2 mm long line feature in four different widths (50, 100, 200, and 400 µm), each positioned at four different angles (0, 15, 30, and 45 degrees). The line features were arranged in a 12.8 mm (X) × 12.8 mm (Y)-sized matrix. The respective Scalable Vector Graphics (SVGs) file was exported and saved as a Joint Photographic Experts Group (JPEG) file. Next, a 10.24 mm (X) × 10.24 mm (Y)-sized plane was created using a polygonal modeling program (Blender, ver. 3.5). The plane that originally consisted of only four vertices (no mesh) was then subdivided six times (i.e., 10.24 mm/64) to create a mesh with a grid width of 0.16 mm, i.e., 160 µm. A subdivision surface modifier was added to the mesh, with the subdivision algorithm set to ‘Simple’ and the viewport level initially set to ‘0’. A texture map was then added to the mesh on the cuboid surface using a displacement modifier (coordinates: UV, direction: normal, strength: −0.25). The test image was projected onto the surface of the meshed plane. In order to enable the textured specimen to be 3D printed, the plane was finally wrapped in a symmetrical polygon with an 18 mm width. The modeling process of the test part is visualized in Figure 1. In order to further increase the mesh resolution without putting too much strain on the hardware, the viewport level was gradually altered within the range of 0 to 5. Screenshots of the meshed surface were taken for each of the six viewport levels. For all features positioned at an angle of 0 degrees, the line widths were measured using an image processing program (ImageJ, release 1.44p) and then plotted against the viewport level on a logarithmic scale.

For each viewport level, the Blender file was exported to the Wavefront (.obj) format and opened in a slicer program (Anycubic PhotonWorkshop), where the print parameters for an LCD resin printer (Anycubic Photon Mono, Shenzhen Anycubic Technology Co., Ltd., Shenzhen, China) were set. Further specifics of the printer kit used in this study are summarized in Table 1. The printer vat was filled with 100 mL of a commercially available resin for elastic 3D prints (RESIONE F80, Dongguan Godsaid Technology Co., Ltd., Dongguan, China). In the slicer program, the cuboid was placed flat at the center of the build envelope and saved as a build file (.pwmo format) that was then uploaded before the print was initiated. After completion of the build job, the part was removed from the build plate and ultrasonically cleaned twice in an ethanol bath (5 min each) using separate containers. After removal from the bath, the rinsed part was blown dry with compressed air and post-cured for 20 min (Asiga Flash, Asiga Pty Ltd, Alexandria, NSW, Australia). Panorama images of the textured samples were taken on a scanning confocal microscope (LEXT OLS4100, Olympus, Tokyo, Japan) at 5× magnification. Using the 20× objective lens on the same instrument, close-up images of the line features positioned at 0 degrees were also taken, through which a line profile was laid to determine the actual feature widths (n = 3) that were plotted against the respective mesh width.

Free stock images with a honeycomb pattern (pattern #7619845, vecteezy.com, accessed on 2 February 2023) and a random line pattern (pattern #335406, vecteezy.com) were downloaded as JPEG files. In the 3D model (cuboid) described above, the image file originally used in the displacement modifier was replaced by the honeycomb and the random texture file, respectively, resulting in differently textured stamps being generated. For maximum accuracy, the viewport levels were set to 5. The models were then exported to the Wavefront format and 3D printed as previously described, applying the optimum cap layer exposure time. The post-cured stamps were then manually coated with a commercially available, oil-based ink for calligraphy/hobbyist applications (Monami Magic). After placing them on a piece of paper, they were loaded with a weight (450 g) for 10 s before the weight and then the stamp were lifted in a vertical movement. Finally, optical microscope images (10×) of the obtained ink patterns were taken.

## 3. Results

### 3.1. Capability Analysis of the Model File Protocol

**Figure 2:**
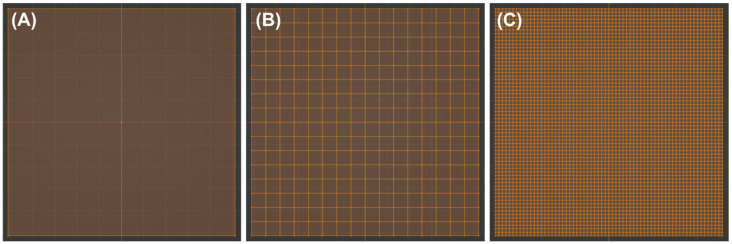
Preparation of the surface to be textured by using subdivisions. (**A**) No subdivisions, (**B**) four subdivisions, and (**C**) six subdivisions. Surface shown in wireframe view.

Before the test image shown in Figure 1 was projected onto the 3D model, the resolution of the accommodating plane had to be increased by gradually reducing the mesh size. To do so, the mesh of the plane was subdivided a total of six times, reducing the mesh width from initially 10.24 mm or 10,240 µm (no subdivisions) to 640 µm (four subdivisions) and further to 160 µm (six subdivisions). The corresponding views of the plane in wireframe view are shown in Figure 2. However, the number of subdivisions in the software used was limited to a maximum of six, so the desired mesh width (resolution) could not yet be achieved with this, as can be seen in Table 2. Based on the mesh width of 160 µm achieved after six subdivisions, a subdivision surface modifier was then added and gradually increased. Even if this was not displayed in the wireframe view, the mesh width could be further reduced to 80 µm (viewport level of 1), 20 µm (level 3), and finally to 5 µm (level 5). The effective mesh size for each resolution is listed in Table 2.

**Figure 3:**
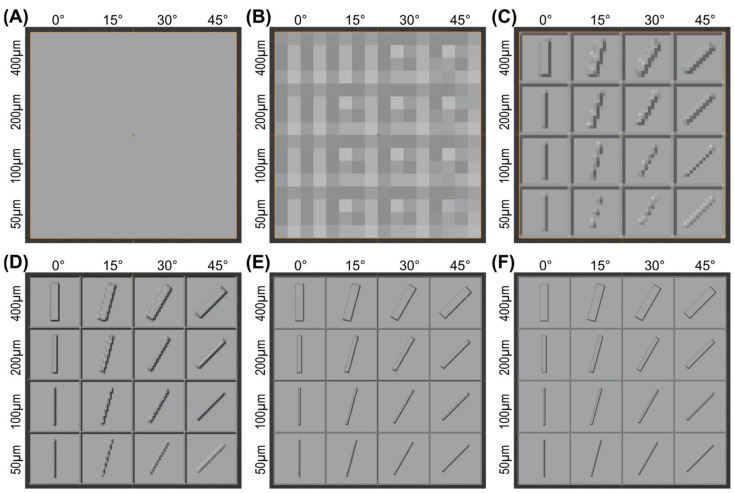
Effect of the mesh width on the appearance of the test part surface with the projected test image. (**A**) No subdivisions, (**B**) subdivided four times, and (**C**) subdivided six times. In addition to the six subdivisions, a subdivision surface modifier was added and set to a viewport level of (**D**) one level, (**E**) three levels, and (**F**) five levels. Surface shown in solid view.

The effective mesh size had a profound influence on the appearance of the projected test image on the test part surface, as shown in Figure 3. Without any modifications to the surface, the test image was simply not recognizable (Figure 3A). After the surface was subdivided four times and a mesh width of 640 µm was achieved, only an extremely coarse and noisy background image was perceptible (Figure 3B). By maximizing the number of divisions to six (160 µm mesh width), the test image was at least recognizable as such, even if it was still very pixelated and blurred (Figure 3C). However, a high-resolution texture could not be created with the subdivisions alone. 

Adding a subdivision surface modifier enabled the effective mesh width to be further reduced, which led to a further improvement in geometric fidelity. After halving the mesh size to 80 µm with just one viewport level, the image became sharper, and at least the horizontally positioned features were rendered accurately (Figure 3D). However, in the rotated features, the stair-stepping effect was very pronounced, especially at 15 degrees. Image quality further improved after the viewport level was increased to three, reducing the mesh size to 20 μm (Figure 3E). Ultimately, however, flawless reproduction of the test image could only be achieved with the smallest mesh size of 5 µm (viewport level of five), as shown in Figure 3F.

**Figure 4:**
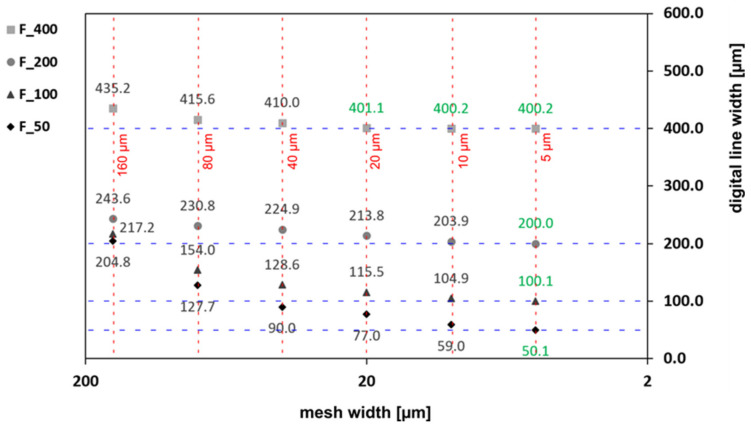
Effect of mesh width on digital line width in the 3D model. Line feature orientation of 0 degrees; nominal line width of 400, 200, 100, and 50 µm.

A more differentiated picture was obtained by measuring out the vertical features and plotting the width data against the different tested mesh widths, as shown in Figure 4. When a coarse (40 µm or larger) mesh was applied, the features in the 3D model were oversized, and the dimensional error was, of course, the more prevalent the thinner the feature was. At a mesh width of 20 µm, at least the thickest line feature (400 µm) was accurately reproduced, with a dimensional error of a mere +0.15 percent. The thinner features, however, could really only be achieved in the 3D model when using the finest of the mesh widths tested here (5 µm). The improvement in reproduction accuracy by reducing the mesh width was, of course, particularly evident in the thinnest feature, which was nominally only 50 µm wide. As an example, when reproducing the 100 µm thin line, the dimensional error amounted to +28.3 percent when using a 50 µm mesh (two viewport levels), but it dropped to just −0.2 percent when switching to a 5 µm mesh (five viewport levels).

### 3.2. Capability Analysis of the 3D Printing Process

**Figure 5:**
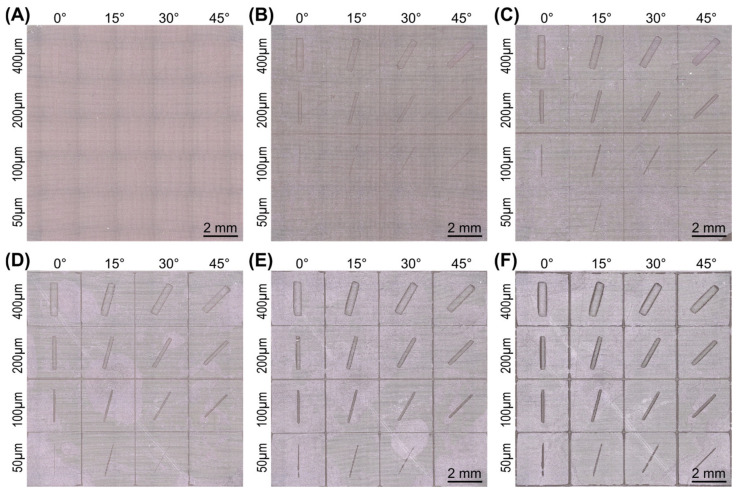
Confocal microscopy images of the textured surface exposed at (**A**) 2.5 s, (**B**) 3.5 s, (**C**) 5.0 s, (**D**) 7.0 s, (**E**) 10.0 s, and (**F**) 15.0 s per surface layer.

The panoramic images taken on a confocal microscope (displayed in Figure 5) confirmed that the exposure time of the textured surface layer had a large impact on the 3D-printed texture. Using the standard exposure time (2.5 s) recommended by the resin manufacturer, the bulk layers of the stamp could be cured without any problems, but only the largest line features (nominally 400 µm thin) were present in the surface layer (Figure 5A). The reason for this lies in the fact that a voxel in a bulk layer is not only exposed to the light dose from the illuminated pixel assigned to it but also to the residual light scattering from up to eight neighboring pixels. This is also known as “cross-talk” [18]. In contrast, the thinner the feature (i.e., the fewer neighboring pixels there are), the less pronounced this effect is, and the net crosslinking energy available in the illuminated pixel remains below the minimum energy required to initiate polymerization.

**Figure 6:**
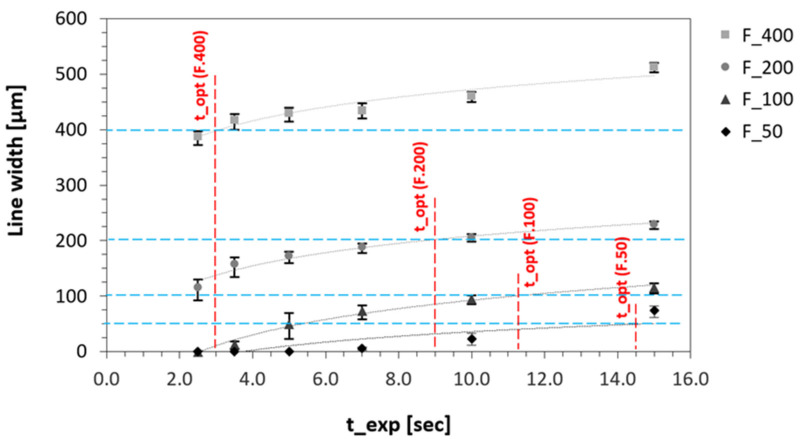
Effect of textured-layer exposure (t_exp) on actual width of the vertical line features and the resulting optimal exposure times (t_opt) for each of the tested feature sizes.

This lack of net crosslinking energy in the voxels of a micro-textured layer can easily be compensated for by overexposure. Here, the overexposure should be higher, the thinner the positive feature is. With the setup used in this study, the 200 µm thin line features were present at a very moderate overexposure of 3.5 s (Figure 5B) and the 100 µm thin features after doubling the surface exposure time to 5 s (Figure 5C). However, the feature, which is only one pixel (50 µm) thin, required an overexposure of 7.5 to 15 s, depending on the positioning (Figure 5D–F). The exact optimal exposure times for each of the four perpendicularly (0 degrees) positioned feature widths tested are shown in Figure 6.

Our results suggest that a positive, protruding microfeature could not or only partially be reproduced (with missing or undersized detail) using the standard exposure time, but the lack of crosstalk in a fine texture can easily be compensated for by carefully adjusting the overexposure of the surface layer, where the optimum should be determined with regard to the thinnest key feature. The method proposed here is not limited to line features and can be transferred to any other feature geometry. This also includes negative features (e.g., microchannels), where, however, significantly shorter surface exposure times or even underexposure can be expected in order to avoid clogging of the fine channels.

### 3.3. Practical Application and Verification

**Figure 7:**
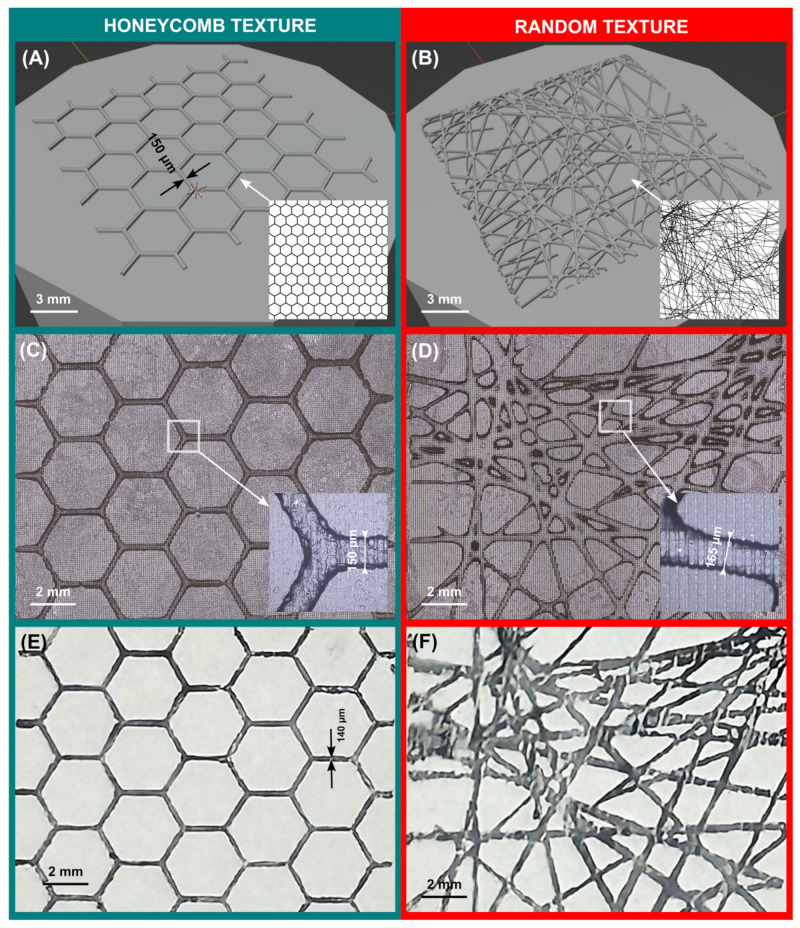
Three-dimensional mesh model of a stamp with (**A**) a regular (honeycomb) texture and (**B**) a random texture, with the imported image shown in the respective inset image. Confocal panoramic image (5×) of the 3D-printed stamp surface for (**C**) the honeycomb texture and (**D**) the random texture, with a magnified view (×20) of a selected detail in each inset image. Optical microscopy image of the ink pattern on paper when using the stamp with (**E**) the honeycomb texture and (**F**) the random texture. Stamping was performed under a load of 450 g for 10 s.

To demonstrate the versatility and applicability of the method presented in this study, a regular and a randomly textured stamp were designed, additively manufactured, and then used in a simple stamping test. Table 3 shows the digital and printing parameters used in the manufacture of the stamps. Screenshots of the stamps obtained by projecting the imported images (honeycomb and random) are shown in Figure 7A,B, respectively. Both stamps contained the desired textures when the textured layers were sufficiently overexposed, as seen in Figure 7C and Figure 7D, respectively.

Determining the optimum exposure time for the randomly textured stamp was challenging, though, because the line thickness varied from 70 µm to 350 µm, so that the optimal overexposure time could not be determined unequivocally. Therefore, we experimented with the previously determined optima t_opt_(F.50), t_opt_(F.100), and t_opt_(F.200), and we settled on t_opt_(F.100) = 11.2 s for both stamps.

Both stamps could be used as intended. The image produced with the honeycomb stamp was high resolution and relatively uniform, as can be seen in Figure 7E. Minor differences in the line thickness (100 to 200 µm) in the ink image were due to a not completely even contact pressure. With regard to the randomly textured stamp, the quality of the inked image was still fair. Fine features were accurately reproduced, but thick strands and the areas where two or more of the strands crossed were slightly oversized (see Figure 7F). This can be attributed to the overexposure set being too high for the thicker lines, or to the contact pressure set being too high. For comparison, the stamps were also printed without overexposure, but the texturing was not present as expected, so no printed image could be obtained.

## 4. Discussion

Surface texturing allows the development of components or substrates with tailored tribological, superhydrophobic, optical, bioactive, surface chemical, or other properties. Since textures are characterized by their geometric features such as shape, dimensions, density, and distribution, finding an effective method to generate the desired surfaces is clearly a key element in realizing potential applications. Although the established micro-cutting and deposition techniques are typically considered for industrial surface texturing [2], 3D printing techniques have recently emerged as an interesting alternative for certain scenarios. Regardless of their geometric features or complexity, 3D printing offers solutions for producing periodic patterns as well as irregularly arranged features, which are quite versatile for the design of functional surfaces.

Inspired by the honeycomb and random textures shown in Figure 7, we see particularly great potential for applications of displacement mapping in texturing bio-active or bio-inspired surfaces. Micropatterning and texturing are of fundamental importance with regard to those biomedical applications, where the main driver is the creation of microenvironments aimed at arranging, manipulating and/or observing cell behavior [19,20,21,22,23,24]. With regard to applications in physiological tissue formation and tissue engineering, natural extracellular matrix-inspired textures are beneficial in controlling cell proliferation [25,26,27,28]. In order to ensure cell compatibility, a whole range of photoactivatable bioresins are now available [29]. The method presented here can also be applied to address anatomically inspired channel networks as a texturing problem, which is highly relevant for microfluidic chips [30,31]. Further applications of nature-inspired surface textures can, of course, also be found outside of microbiology, such as for basic research in surface science [32,33], stamping and patterning tools [34,35], high-tech surfaces [36], or optoelectronic surfaces [37,38].

The results obtained in this study demonstrate how an approach originally designed for computer graphics can also be useful as a fast and highly flexible texturing tool in a technical context. Compared to conventional micromolding, micromilling or laser surface texturing, the method described here has clear advantages in terms of greater design flexibility, shorter setup times, and much cheaper acquisition costs for the required hardware, which is of particular interest when prototyping and producing small batch sizes. The biggest disadvantage of this method may be that the textured components always consist of a photopolymerized resin, which is simply unsuitable for many applications. However, displacement mapping can also be combined with other 3D printing techniques such as selective laser sintering (SLS) or selective laser melting (SLM), which extends the range of available materials to ceramics and metals [39,40]. For textures where a resolution in the nanometer range is required, 3D printers based on two-photon polymerization [41] or multiphoton lithography [42] are potentially needed, which may provide an interesting alternative to conventional high-resolution techniques such as laser surface texturing or photolithography.

## 5. Conclusions

This study demonstrates how displacement mapping can be used in combination with resin 3D printing as a fast and highly flexible surface texturing technique. In addition, a methodology was developed to predict the dimensional capability of the model file protocol and a given 3D printing setup. We were able to create protruding microtextures on a resin 3D printer with a resolution (XYZ) of approximately 50 microns when (1) the resolution of the corresponding mesh model was increased to 5 microns and (2) the textured cap layer was overexposed by a factor of 4.5 compared to the bulk exposure time. However, the level of overexposure must be determined individually for each texture because it depends heavily on the texture characteristics (geometry and size) as well as the printer setup (light source, resolution, and resin). As an example of a practical application, functional stamps with a regularly and randomly textured surface were produced. Apart from patterning tools, we see great potential for the method presented here in the creation of tailor-made components with bio-inspired and bio-active surfaces, in particular for studies of cell behavior, the control of cell proliferation, or in microfluidic chips.

## Figures and Tables

**Table 1:** Printer specifications and print settings used in this study.

Printer Specifications	Print Settings
3D printer model: Anycubic Photon Mono	Layer thickness (Z): 50 µm
Light source: 45-Watt monochrome	Burn-in layers: 3
Wavelength: 405 nm	Exposure (burn-in): 30 s
Light patterning unit: LCD shadow mask	Exposure (bulk): 2.5 s
Resolution: 2560 × 1620 pixels (2K)	Exposure (cap): From 2.5 to 15.0 s
Pixel size (XY): 51 µm	Anti-aliasing: 2

**Table 2:** Effective mesh size at each resolution.

Number of Divisions	Viewport Levels	Mesh Size (µm)
0	0	10,240
1	0	5120
2	0	2560
3	0	1280
4	0	640
5	0	320
6	0	160
6	1	80
6	2	40
6	3	20
6	4	10
6	5	5

**Table 3:** Parameters used in the manufacture of the stamps.

Parameter	Setting
Number of divisions:	6
Viewport levels:	5
Mesh size:	5 µm
Pixel size (XY):	51 µm
Layer thickness (Z):	50 µm
Exposure time (bulk layers):	2.5 s
Exposure time (textured layers):	11.2 s

## Data Availability

The raw data supporting the conclusions of this article will be made available by the corresponding author on request.
